# *Yersinia pseudotuberculosis* ST42 (O:1) Strain Misidentified as *Yersinia pestis* by Mass Spectrometry Analysis

**DOI:** 10.1128/genomeA.00435-14

**Published:** 2014-06-12

**Authors:** Patrick Gérôme, Philippe Le Flèche, Yann Blouin, Holger C. Scholz, François M. Thibault, Françoise Raynaud, Gilles Vergnaud, Christine Pourcel

**Affiliations:** a Service de Biologie Médicale, HIA Desgenettes, Lyon, France; b University Paris-Sud, Institut de Génétique et Microbiologie, Orsay, France; c Centre National de la Recherche Scientifique (CNRS), Orsay, France; d Department of Analytical Microbiology, DGA, MNRBC, Vert le Petit, France; e Bundeswehr Institute of Microbiology, Munich, Germany; f Institut de Recherche Biomédicale des Armées (IRBA), Brétigny, France; g Direction Générale de l'Armement (DGA), Bagneux, France

## Abstract

We report here the draft sequence of strain CEB14_0017, alias HIAD_DUP, recovered from a human patient and initially identified as *Yersinia pestis* by mass spectrometry analysis. Genotyping based on tandem repeat polymorphism assigned the strain to *Yersinia pseudotuberculosis* sequence type 42 (ST42). The total assembly length is 4,894,739 bp.

## GENOME ANNOUNCEMENT

A 50-year-old male window cleaner was admitted to the infectious disease unit because of a 2-month history of asthenia and weight reduction of 15 kg. During the preceding 2 weeks, he developed fever between 38 and 39°C, with chills and abdominal discomfort that were accompanied by diarrhea. He also reported polyarthralgia and low back pain. He had a history of zoster infection 2 months and 5 years earlier. On admission, the patient had a fever (38.5°C), a heart rate of 102 bpm, and a blood pressure of 110/65 mm Hg. His body mass index (BMI) was 15.6, and the other physical findings were unremarkable. He did not present with signs of severe sepsis.

Blood tests revealed anemia (hemoglobin level of 95 g/liter), a platelet count of 115 × 10^9^/liter, a white blood cell count of 4.8 × 10^9^/liter (64% polymorphonuclear leukocytes, 19% lymphocytes), and increased serum C-reactive protein (100 mg/liter) and serum procalcitonin (4.5 ng/ml). Biochemical tests showed altered levels of creatinine (118 mmol/liter), elevated aspartate aminotransferase (150 UI/liter) and alanine aminotransferase (65 UI/liter), and hyponatremia (128 mmol/liter). An HIV test was positive (viremia, 556,000 copies/ml; CD4^+^ cell count, 80/mm^3^). A thoracic and abdominal computed tomography scan revealed the presence of a Douglas pouch effusion without colon wall thickening.

Antimicrobial treatment with ceftriaxone (2 g/day intravenously for 5 days) and ciprofloxacin (400 mg/day orally for 10 days) led to a total remission of symptoms after 5 days.

A bipolar Gram-negative rod was isolated from blood drawn on admission using a BacT/Alert (bioMérieux, Marcy l’Étoile, France). This nonmotile, oxidase-negative, facultative aerobic bacillus was able to grow on Trypticase soy agar (TSA), blood agar, and chocolate agar plates within 18 h.

The identification of the isolate was performed by matrix-assisted laser desorption ionization–time of flight mass spectrometry (MALDI-TOF MS) using the MALDI Biotyper (Bruker Daltonique, Wissembourg, France). The closest match was to *Yersinia pestis* (score, 2.388 [score of >2.0 is considered a species-level identification]). Phenotypic identification using the Vitek 2 GN system (bioMérieux, Marcy l’Étoile, France) gave *Yersinia pseudotuberculosis* as a best match, with a probability of 99%.

The multilocus variable-number tandem-repeat analysis (MLVA) (1, 2) 25-loci code of strain CEB14_0017 is 6-7-5-ND-6-ND-10-11-10-7-8-8-7-6-5-5-1-8-5-9-5-4-4-5-6, which corresponds to *Y. pseudotuberculosis* sequence type 42 (ST42) strains (3; G. Vergnaud, P. Le Flèche, H. C. Scholz, and C. Pourcel, unpublished data). Whole-genome sequencing was performed using MiSeq (Illumina, Inc., San Diego, CA) by generating 250-bp paired-end reads from libraries with an insert size of 500 bp. Two million reads passed the Illumina quality filters. One million reads were *de novo* assembled using Velvet, with a *k*-mer value of 31, and Ray, with *k* of 29, accessible within BioNumerics 7.1 (Applied Maths, Belgium). The coding sequences (CDSs) were predicted using BioNumerics. The largest contig is 360,989 bp. The total length of the 128 contigs >1 kb is 4,894,739 bp, comprising 4,229 predicted CDSs. The average G+C content of the draft genome is 47.4%.

### Nucleotide sequence accession numbers. 

This whole-genome shotgun (WGS) project has been deposited in the European Nucleotide Archive at EMBL-EBI under accession no. CCBI010000001 to CCBI010000128 within WGS project CCBI000000000.
